# Spatiotemporal characterization of a distinct nectin-3^+^ cell population in the developing and adult mouse retina

**DOI:** 10.1038/s41598-026-56219-9

**Published:** 2026-06-08

**Authors:** Agnieszka Lukomska, Patrycja Kieszek, Natalia Buczek, Mariusz Z. Ratajczak, Magdalena Kucia

**Affiliations:** 1 Center for Preclinical Studies and Technology, Department of Regenerative Medicine, Medical University of Warsaw, 1b Banacha St, Warsaw, 02-097 Poland; 2 Faculty of Pharmacy, Medical University of Warsaw, 1 Banacha Street, Warsaw, 02-091 Poland; 3 Stem Cell Institute at Graham Brown Cancer Center, University of Louisville, 500 S. Floyd Street, Rm. 107, Louisville, KY 40202 USA

**Keywords:** Nectin-3, Molecular signature, Retinal development, Surface marker, Biological techniques, Cell biology, Developmental biology, Neuroscience, Stem cells

## Abstract

While Nectin-3 is a recognized marker for maintaining stem and progenitor “side populations” in non-neural epithelia, its role and distribution within the mammalian retina remain largely uncharacterized. Identifying such rare populations is a critical step toward unlocking the regenerative potential of the retina after injury. This study provides a comprehensive spatiotemporal characterization of Nectin-3^+^ cells from embryonic development through adulthood. Using fluorescence-activated cell sorting, a distinct Nectin-3^+^ population was identified across all examined stages, representing 1.27% ± 0.24 of viable cells at E18, peaking at P5 (3.62% ± 0.58), and persisting in the adult retina (1.43% ± 0.14). At this mature stage, triple-positive (Nectin-3^+^/CD117^+^/Sca-1^+^) cells displayed a robust molecular signature defined by the significant up-regulation of *Chx10*,* Nestin*,* Pax6*,* Rax*, and the RGC marker *Rbpms*, despite a lack of synaptic transcript (*Syp*) enrichment. Morphological analysis via confocal microscopy demonstrated that Nectin-3^+^ cells undergo dynamic changes during development. In the adult retina, these cells showed colocalization with RBPMS, while remaining distinct from the Müller glial scaffold. Principal Component Analysis highlighted that adult cells retain transcriptional features typically associated with early development, indicating a specialized neuronal identity that is molecularly distinct from the surrounding retinal environment. By defining this fraction, we provide a practical tool for studying retinal diversity and a foundation for investigating the potential regenerative capacity of these rare cells.

**Supplementary Information:**

The online version contains supplementary material available at 10.1038/s41598-026-56219-9.

## Introduction

Irreversible vision loss remains a major global health challenge, with glaucoma and optic neuropathies among the leading causes of blindness worldwide^1,2^. While the mammalian retina is traditionally characterized by limited regenerative capacity, recent advances suggest a higher degree of cellular heterogeneity than previously recognized. A significant barrier to understanding retinal homeostasis and potential repair mechanisms is the lack of reliable surface markers to prospectively identify and isolate rare cell populations with immature molecular profiles, particularly in the adult retina, where the persistence of cells with such features remains poorly defined.

Cell surface adhesion proteins of the nectin family, including Nectin-3 (also known as PVRL3, CD113, or PRR3), are Ca^2+^-independent immunoglobulin-like molecules that regulate fundamental processes such as adhesion, migration, and polarization^3–5^. By engaging in both homophilic and heterophilic interactions, nectins contribute to the formation of intercellular junctions crucial for tissue organization^6,7^. Specifically within the eye, Nectin-3 heterophilic trans-interactions are essential for establishing the apex-apex adhesion between ciliary epithelia, as its deficiency leads to microphthalmia and impaired ciliary body morphogenesis^8^. While Nectin-3 has been implicated in maintaining stem/progenitor populations in non-neural epithelia—such as the side population (SP) of rabbit limbal epithelial cells^9,10^ its specific role and distribution within the mammalian retina remain largely uncharacterized.

While single-cell transcriptomics has delineated various transcriptional states in development and human tissue^11^, a major limitation remains the lack of reliable surface markers for the prospective isolation of cells with such profiles from the adult mammalian retina. Most current approaches rely on *post hoc* signatures or indirect markers like Sca-1, CD117, and side-population analysis, which often lack the specificity required for precise experimental or translational applications. Consequently, the ability to directly enrich populations with immature molecular features from the adult retina remains a critical unmet need.

The present study demonstrates that cells expressing Nectin-3 as a surface marker exhibit a distinct transcriptional profile characterized by the elevated expression of genes typically associated with early neural development. Consequently, Nectin-3 represents a promising tool for the prospective isolation and further investigation of these molecularly unique cell fractions. Building on this, the aim of the present study was to map the spatiotemporal distribution of Nectin-3^+^ cells and examine the persistence of a specific developmental gene signature within these populations across both the developing and adult retina.

## Results

To characterize retinal cell populations, we analyzed and sorted cells based on surface marker expression at three developmental stages: E18, P5, and adulthood. Our analysis identified Nectin-3^+^ cells as a persistent subpopulation across all examined ages. Subsequent gene expression analysis revealed that while Nectin-3^+^ cells are enriched for early retinal transcripts (*Nestin*,* Pax6*,* Chx10*,* Rax*,* and Sox2*), the highest expression levels were specifically associated with subpopulations co-expressing CD117 (c-kit) and/or Sca-1. These molecular profiles varied according to the developmental stage, suggesting that Nectin-3 serves as a primary marker for a heterogeneous population with age-dependent sub-phenotypes.

### Prospective isolation of the target cell populations

**Fig. 1:**
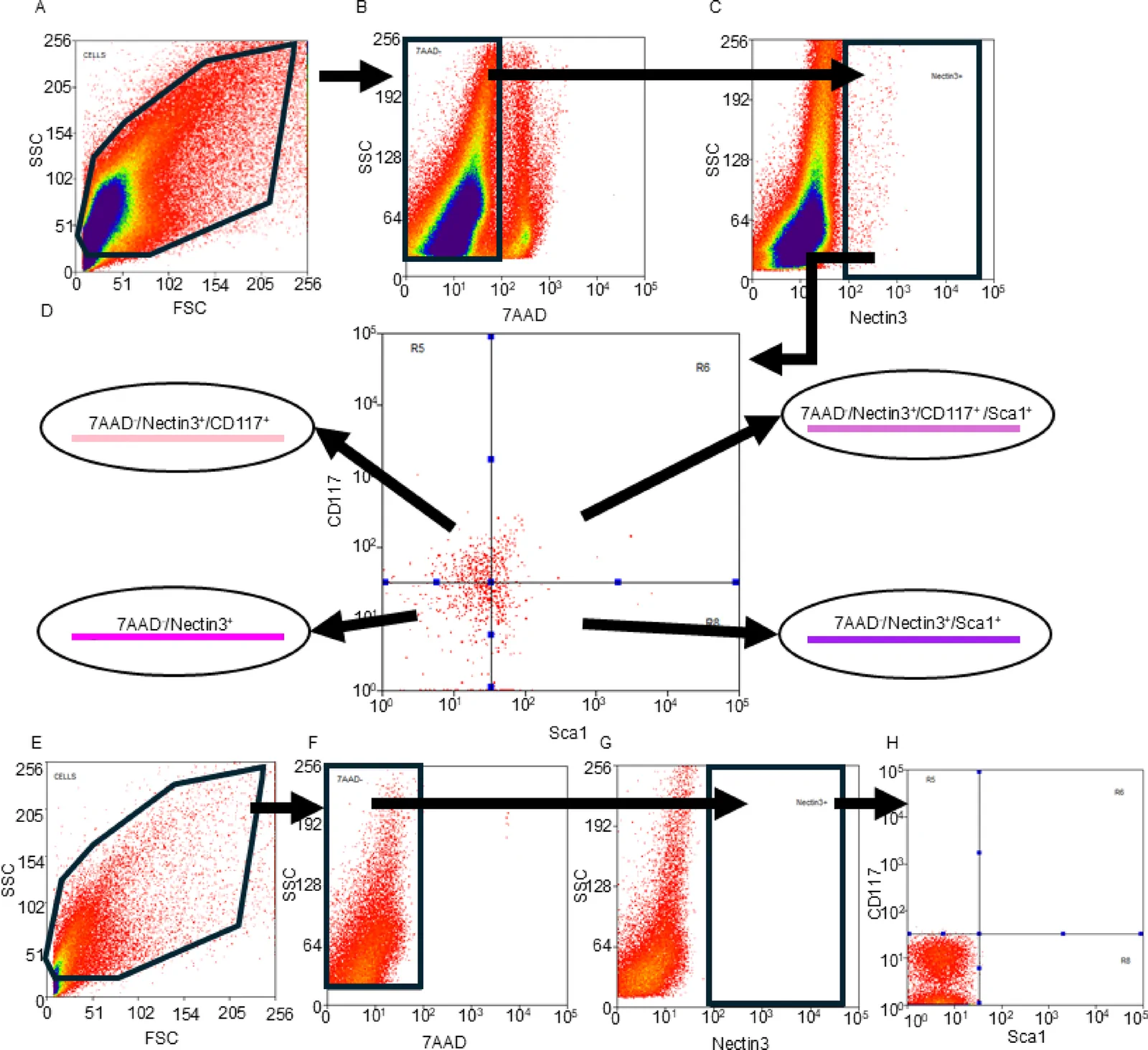
Gating strategy for the isolation of Nectin-3^+^ cell subpopulations from the retina. (**A**) Representative flow cytometry plot showing the initial gating based on forward scatter (FSC) and side scatter (SSC) to exclude cellular debris. (**B**) Subsequent gating on a 7-AAD vs. SSC plot to select for viable (7-AAD⁻) cells and exclude dead cells. (**C**) Viable cells were then gated for Nectin-3^+^ expression. (**D**) The Nectin-3^+^ population was further subdivided using a bivariate plot of CD117 versus Sca-1 expression to isolate four distinct subpopulations: Nectin-3^+^/CD117⁻/Sca1⁻, Nectin-3^+^/CD117^+^, Nectin-3^+^/Sca1^+^, and Nectin-3^+^/CD117^+^/Sca1^+^. The unstained sample shown in (**E**–**H**) served as a negative control for fluorescence thresholds. All plots are representative of the gating strategy used for all biological replicates and developmental stages investigated (E18, P5, and adult).

We employed a sequential gating strategy to isolate specific progenitor-like cell populations from retinal cell suspensions using fluorescence-activated cell sorting (FACS). The initial gating was performed on a forward scatter (FSC) versus side scatter (SSC) plot to include all cells and exclude debris (Fig. 1A). Subsequently, viable cells were selected by gating on the 7-aminoactinomycin D (7-AAD) negative population to exclude dead cells (Fig. 1B). From this viable cell population, we identified the Nectin-3^+^ cells (Fig. 1C). Finally, the Nectin-3^+^ population was further subdivided into four distinct subpopulations based on the expression of CD117 and Sca-1 using a bivariate plot: Nectin-3^+^/CD117⁻/Sca1⁻, Nectin-3^+^/CD117^+^, Nectin-3^+^/Sca1^+^, and Nectin-3^+^/CD117^+^/Sca1^+^ (Fig. 1D). These procedures allowed for the prospective isolation of the target cell populations from each developmental stage for further downstream analysis.

### E18 retina

**Fig. 2:**
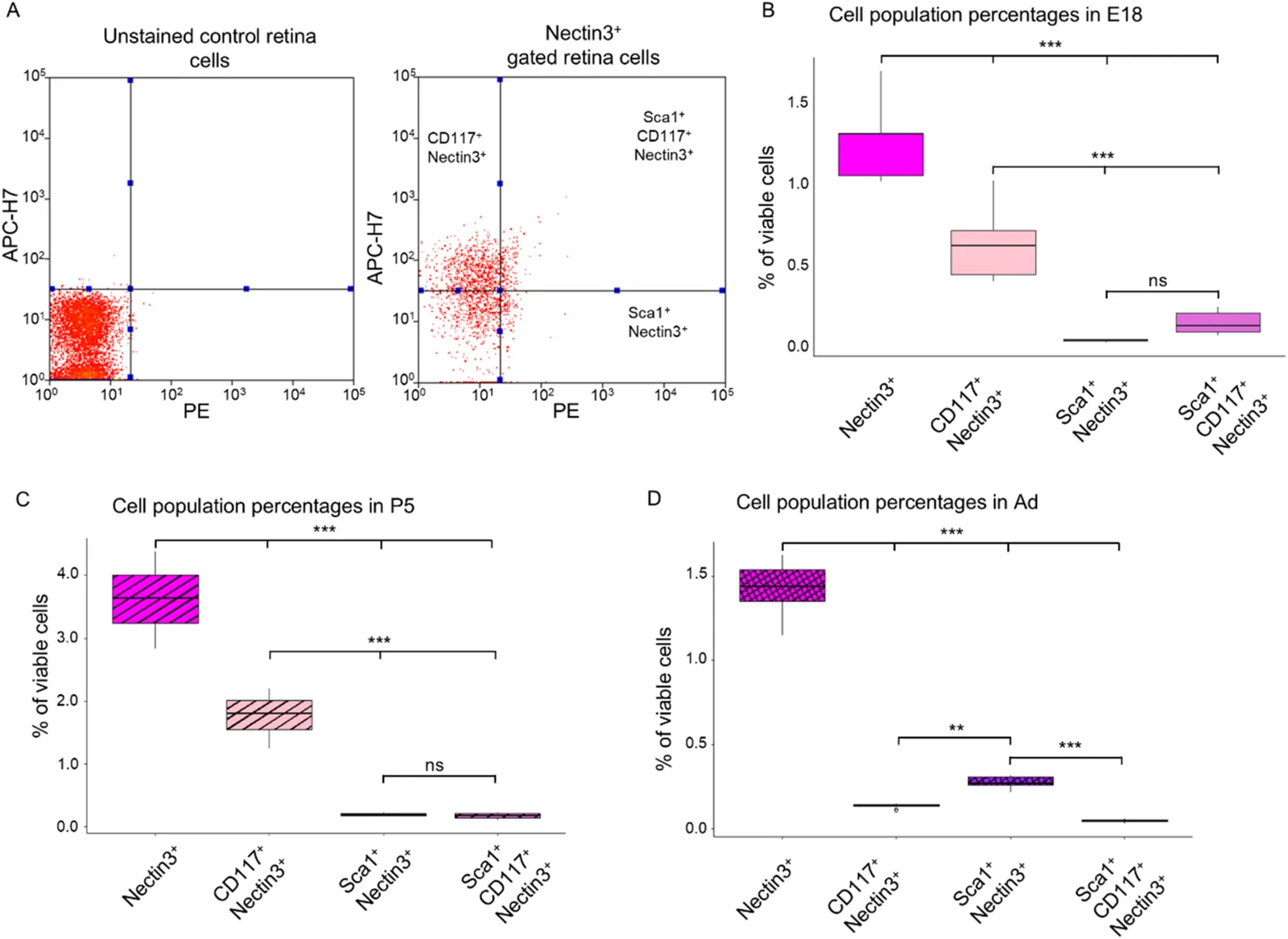
Quantitative analysis of Nectin-3^+^ retinal cell subsets across developmental stages. (**A**) Representative flow cytometry plots showing the gating strategy used to identify Nectin-3^+^ cells and their subpopulations based on CD117 and Sca-1 expression in dissociated mouse retinas. An unstained sample is shown as a negative control. (**B**–**D**) Quantitative analysis of the indicated cell subpopulation frequencies, expressed as a percentage of total viable (7-AAD⁻) cells at E18 (**B**), P5 (**C**), and adult (**D**) stages. Data are presented as box plots (*n* = 3 independent biological replicates). Statistical significance between subpopulation frequencies within each age group was determined by one-way ANOVA followed by Tukey’s post-hoc test (*p < 0.05, **p < 0.01, ***p < 0.001).

Analysis of retinal cell suspensions from E18 embryos identified a distinct Nectin-3^+^ population, which constituted 1.27% (± 0.24%) of total viable cells. This population was further subdivided based on the co-expression of CD117 and Sca-1. The Nectin-3^+^/CD117^+^ subpopulation accounted for 0.64% (± 0.22%) of total cells, while the Nectin-3^+^/Sca-1^+^ and the triple-positive Nectin-3^+^/CD117^+^/Sca-1^+^ subpopulations were less frequent, representing 0.04% (± 0.01%) and 0.15% (± 0.07%) of total cells, respectively (Fig. 2B).

**Fig. 3:**
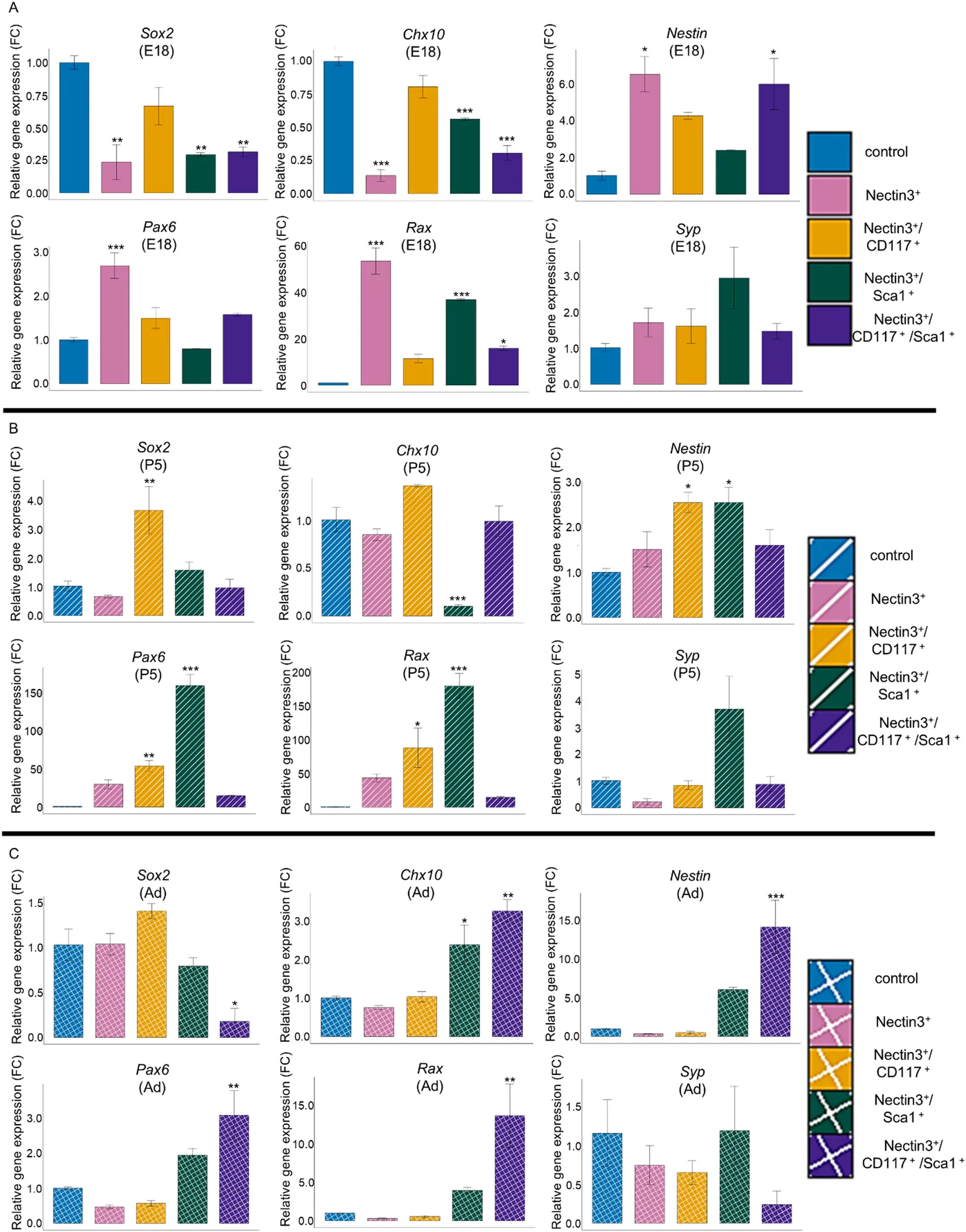
Developmental stage-specific gene expression signatures of Nectin-3^+^ cell subpopulations. Relative mRNA expression (fold change) of progenitor-associated markers (*Sox2*,* Chx10*,* Nestin*,* Pax6*, and *Rax*) and the synaptic marker (*Syp*) in sorted cell fractions at (**A**) E18, (**B**) P5, and (**C**) Adult stages. In each panel, expression levels for individual genes are shown across the isolated subpopulations. Values were normalized to *Gapdh* and are presented relative to an age-matched unsorted retinal control. Data are presented as mean ± SEM (*n* = 3). Statistical significance compared to the respective unsorted control was determined by one-way ANOVA followed by Tukey’s post-hoc test (*p < 0.05, **p < 0.01, ***p < 0.001). Colors indicate specific cell subpopulations as indicated in the legend. Fill patterns indicate developmental stages: solid bars (E18), striped bars (P5), and crosshatched bars (Adult).

Quantitative PCR analysis of sorted E18 subpopulations revealed distinct transcriptional profiles for key progenitor-associated markers (Fig. 3A). The Nectin-3^+^ single-positive population was characterized by a significant enrichment of *Nestin*, *Pax6*, and *Rax* compared to the unsorted E18 control. Similarly, the Nectin-3^+^/Sca-1^+^ and Nectin-3^+^/CD117^+^/Sca-1^+^ fractions both exhibited increased expression of *Rax*. Notably, the Nectin-3^+^/CD117^+^/Sca-1^+^ triple-positive subpopulation also demonstrated significantly elevated levels of *Nestin*, further distinguishing its molecular signature during embryonic development. In contrast, no significant differences in synaptophysin (*Syp)* expression were observed across any of the Nectin-3^+^ fractions at this stage, indicating a lack of synaptic transcript enrichment relative to the unsorted control.

### P5 retina

At P5, the Nectin-3^+^ population was more abundant, representing 3.62% (± 0.58%) of the total neonatal retinal cell suspension. Further characterization of this population revealed that Nectin-3^+^/CD117^+^ cells were the most frequent subpopulation, accounting for 1.76% (± 0.35%) of total cells. The Nectin-3^+^/Sca-1^+^ and the triple-positive Nectin-3^+^/CD117^+^/Sca-1^+^ subpopulations were present at lower frequencies, constituting 0.19% (± 0.02%) and 0.18% (± 0.04%) of total cells, respectively (Fig. 2C).

Gene expression analysis of these neonatal subpopulations also showed distinct profiles (Fig. 3B). The Nectin-3^+^/CD117^+^ cells were characterized by increased expression of *Sox2*, *Nestin*, *Pax6*, and *Rax*. Similarly, the Nectin-3^+^/Sca-1^+^ population showed elevated expression of *Nestin*, *Pax6*, and *Rax*. Consistent with the findings at E18, no significant differences in *Syp* expression were observed across any of the Nectin-3^+^ neonatal fractions compared to the unsorted control.

### Adult retina

In retinal cell suspensions from adult mice, several distinct Nectin-3^+^ subpopulations were identified. The most prominent of these consisted of cells expressing only the Nectin-3 marker, which accounted for 1.43% (± 0.14%) of total viable cells. The double-positive Nectin-3^+^/Sca-1^+^ and Nectin-3^+^/CD117^+^ subpopulations represented 0.28% (± 0.03%) and 0.13% (± 0.01%) of total cells, respectively. The triple-positive Nectin-3^+^/CD117^+^/Sca-1^+^ subpopulation was the least frequent, at 0.05% (± 0.01%) (Fig. 2D).

Gene expression analysis of the adult retina revealed highly specific transcriptional profiles across the isolated fractions (Fig. 3C). Notably, the triple-positive (Nectin-3^+^/CD117^+^/Sca-1^+^) subpopulation displayed a robust molecular signature characterized by the enrichment of *Chx10*,* Nestin*,* Pax6*, and *Rax*. Furthermore, similar to the embryonic and neonatal stages, *Syp* expression levels did not show significant differences compared to the age-matched unsorted control across any of the adult Nectin-3^+^ fractions.

**Fig. 4:**
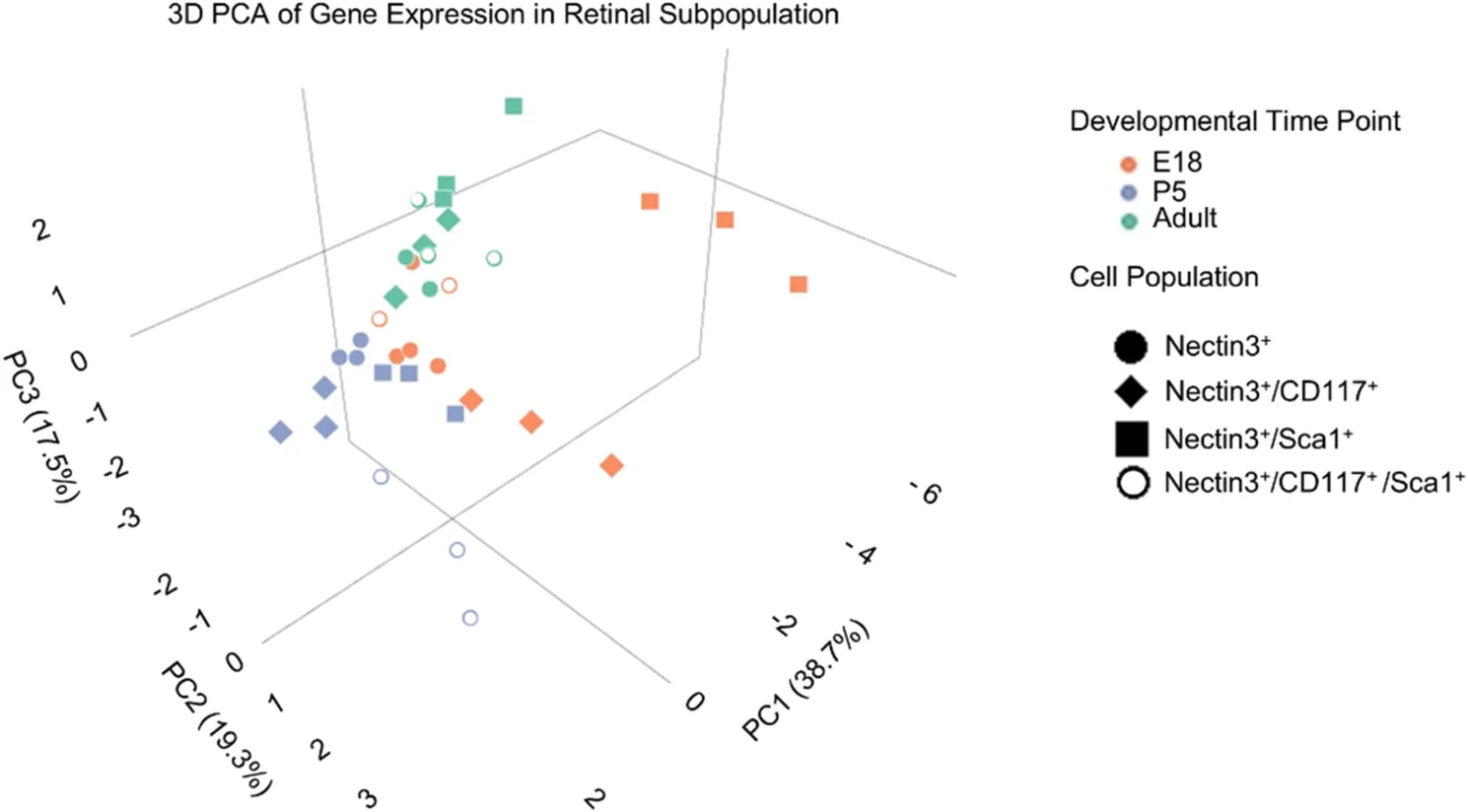
Principal component analysis (PCA) of gene expression in Nectin-3^+^ cell subpopulations. A three-dimensional PCA plot showing the relationships among the Nectin-3^+^ subpopulations based on the expression of eight genes (*Nestin*,* Sox2*,* Pax6*,* Chx10*,* Rax*,* Rbpms*,* Glul*, and *Syp*). The PCA was performed on the mean expression data, with each point representing a distinct cell sample. The plot displays the first three principal components (PC1, PC2, and PC3), which accounted for 38.6%, 19.3%, and 17.4% of the total variance, respectively. Points are colored by developmental time point (embryonic, postnatal day 5, and adult) and shaped by their specific cell markers, allowing for clear visualization of the relationships between subpopulations.

To contextualize these findings within the developmental landscape, we performed a three-dimensional Principal Component Analysis (PCA) based on the expression of eight genes, including those associated with retinal development (*Nestin*, *Sox2*, *Pax6*, *Chx10*, *Rax*), the retinal ganglion cell (RGC) marker *Rbpms*, the glial marker *Glul*, and the synaptic marker *Syp* (Fig. 4). The first three principal components collectively accounted for 75.3% of the total variance (PC1: 38.6%; PC2: 19.3%; PC3: 17.4%).

The PCA revealed a high degree of transcriptional similarity between the adult Nectin-3^+^ subpopulations and the embryonic (E18) populations, which clustered in close spatial proximity. This suggests that adult Nectin-3^+^ cells maintain a molecular profile characterized by the persistence of transcripts typically enriched during early retinal development. In contrast, the P5 Nectin-3^+^/CD117^+^ and Nectin-3^+^/Sca-1^+^ subsets appeared as distinct outliers along the principal axes, likely reflecting a unique, developmentally dynamic state during this window of postnatal expansion. By integrating markers of both developmental identity and functional maturation, the PCA provides a visualization of a consistent molecular trajectory from E18 through adulthood, while simultaneously highlighting the transcriptional distance between these cells and more terminally differentiated or synaptically mature states.

### Localization and morphology of nectin-3^+^ cells in the developing and adult retina

**Fig. 5:**
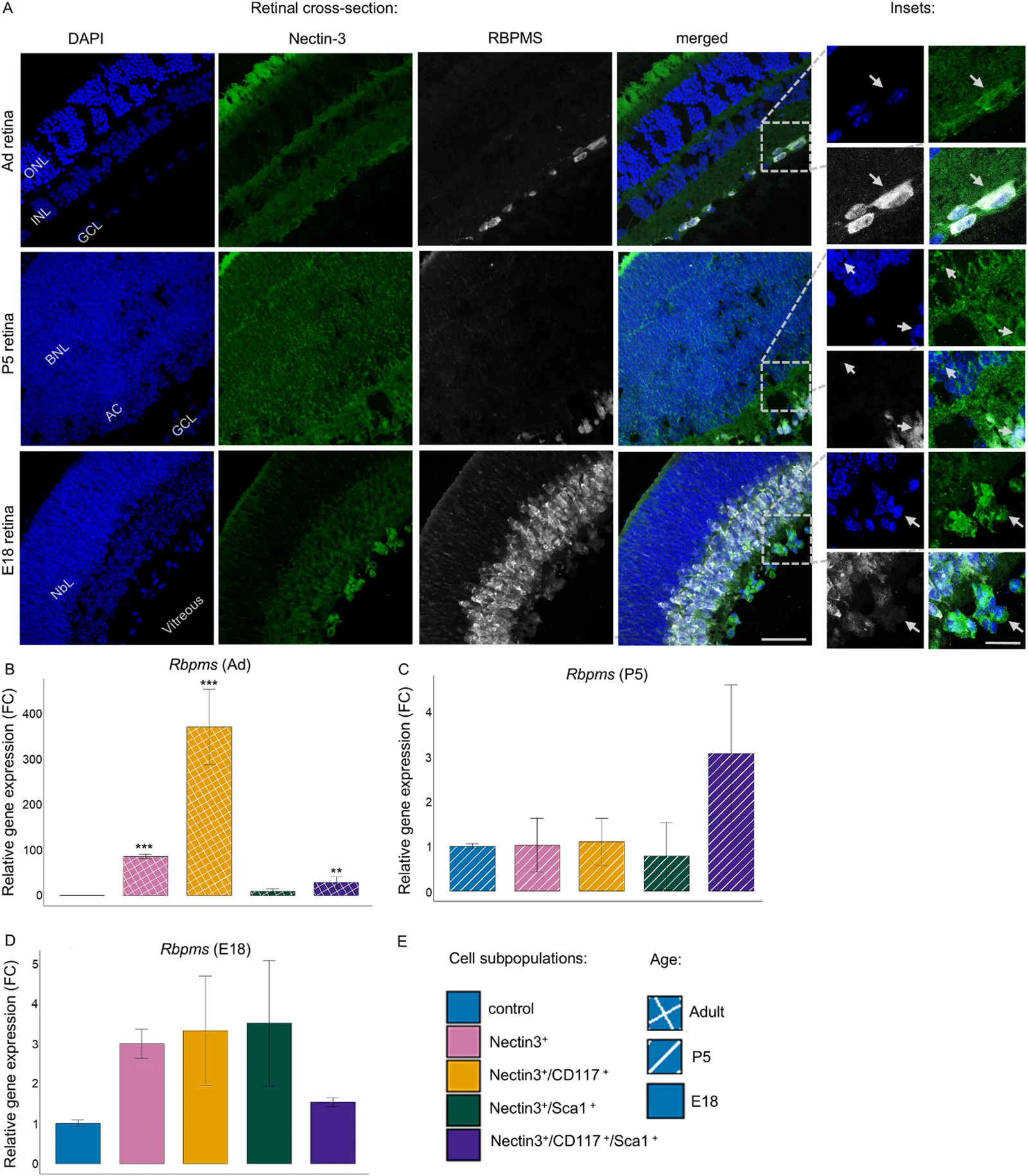
Spatiotemporal localization of Nectin-3^+^ cells and transcriptional enrichment of the RGC marker *Rbpms*. (**A**) Representative confocal microscopy images of retinal cross-sections from adult (top), P5 (middle), and E18 (bottom) mice. For each stage, the panel sequence from left to right shows: DAPI nuclear stain (blue), Nectin-3 (green), RBPMS (grayscale), and the merged image. High-magnification insets are provided at the far right. Retinal layers are labeled on the DAPI images: Adult (ONL, outer nuclear layer; INL, inner nuclear layer; GCL, ganglion cell layer); P5 (BNL, basal neuroblastic layer; AC, emerging amacrine cell layer; GCL); and E18 (NbL, neuroblastic layer; Vitreous). In the merged insets, arrows indicate Nectin-3^+^ cells. Scale bars: 50 μm (main images) and 20 μm (insets). (**B**–**D**) Gene expression levels for *Rbpms* across the isolated Nectin-3^+^ subpopulations at all developmental stages. Values were normalized to *Gapdh* and are presented relative to an age-matched unsorted retinal control. (**E**) Colors indicate specific cell subpopulations as defined in the legend. Fill patterns indicate developmental stages: solid bars (E18), striped bars (P5), and crosshatched bars (Adult). Data are presented as mean ± SEM (*n* = 3). Statistical significance compared to the respective unsorted control was determined by one-way ANOVA followed by Tukey’s post-hoc test (*p < 0.05, **p < 0.01, ***p < 0.001).

**Fig. 6:**
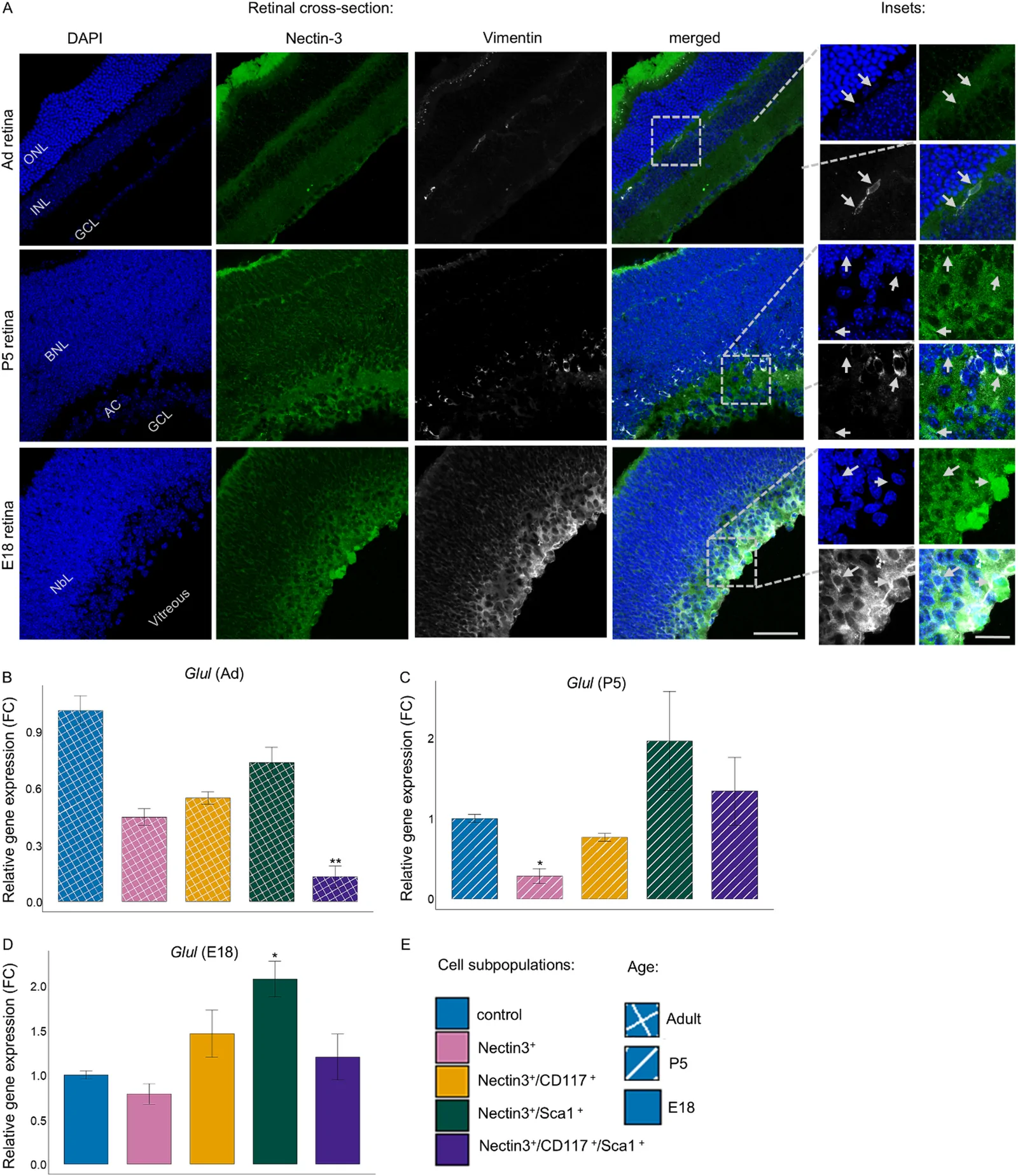
Spatiotemporal localization of Nectin-3^+^ cells and transcriptional expression of the Müller glia marker *Glul*. (**A**) Representative confocal microscopy images of retinal cross-sections from adult (top), P5 (middle), and E18 (bottom) mice. For each stage, the panel sequence from left to right shows: DAPI nuclear stain (blue), Nectin-3 (green), Vimentin (grayscale), and the merged image. High-magnification insets are provided at the far right. Retinal layers are labeled on the DAPI images: Adult (ONL, outer nuclear layer; INL, inner nuclear layer; GCL, ganglion cell layer); P5 (BNL, basal neuroblastic layer; AC, emerging amacrine cell layer; GCL); and E18 (NbL, neuroblastic layer; Vitreous). In the merged insets, arrows indicate Nectin-3^+^ cells. Scale bars: 50 μm (main images) and 20 μm (insets). (**B**–**D**) Gene expression levels for *Glul* across the isolated Nectin-3^+^ subpopulations at all developmental stages. Values were normalized to *Gapdh* and are presented relative to an age-matched unsorted retinal control. (**E**) Colors indicate specific cell subpopulations as defined in the legend. Fill patterns indicate developmental stages: solid bars (E18), striped bars (P5), and crosshatched bars (Adult). Data are presented as mean ± SEM (*n* = 3). Statistical significance compared to the respective unsorted control was determined by one-way ANOVA followed by Tukey’s post-hoc test (*p < 0.05, *p < 0.01).

To investigate the in situ localization and morphology of Nectin-3^+^ cells, we performed immunofluorescence staining on retinal cross-sections across three developmental stages. This analysis confirmed the presence of Nectin-3^+^ cells at E18, P5, and in the adult retina, revealing distinct morphological and laminar characteristics at each stage (Figs. 5A and 6A).

In the adult tissue, Nectin-3 immunoreactivity was observed within the extracellular matrix (ECM) between the ganglion cell layer (GCL) and the inner nuclear layer (INL), as well as between the INL and the outer nuclear layer (ONL). The most intense fluorescence signal was detected within the inner and outer segment (IS/OS) layers. At P5, Nectin-3 signal intensity was markedly higher than at other stages, spanning the entire retinal thickness, particularly within the neuroblastic layer (NbL). Given the low cellular yields obtained via FACS at this stage, we interpret this widespread P5 signal as predominantly extracellular. At E18, the signal was primarily concentrated within the forming GCL, exhibiting a clearly defined cellular localization. We distinguish between membrane-associated (cellular) and diffuse extracellular Nectin-3 signal based on morphology, co-localization, and FACS-derived cellular enrichment, supporting the interpretation that the sorted Nectin-3^+^ population reflects bona fide cells rather than extracellular debris.

To determine the identity of these cells, we performed co-staining with the RGC marker RBPMS (Fig. 5A). In the adult retina, cellular Nectin-3 signal clearly colocalized with a subset of RBPMS-positive cells within the GCL (Fig. 5A, upper right panel, insets). At P5, we observed a mixed population within the developing GCL, including double-positive cells as well as Nectin-3^+^ cells that were negative for RBPMS, particularly within the emerging amacrine cell (AC) layer (Fig. 5A, middle right panel, insets). At E18, Nectin-3^+^ cells were prominently localized near the vitreous surface; these cells exhibited a weak but detectable RBPMS signal, while strongly RBPMS-positive cells were generally situated deeper within the retina closer to the NbL (Fig. 5A, bottom right panel, insets).

These imaging findings were corroborated by qPCR analysis of sorted subpopulations (Fig. 5B). In the adult retina, all investigated Nectin-3^+^ fractions showed a significant enrichment of the *Rbpms* transcript. Specifically, the Nectin-3^+^ single-positive fraction exhibited a 86 ± 4.9 FC, while the Nectin-3^+^/CD117^+^ and triple-positive (Nectin-3^+^/CD117^+^/Sca-1^+^) fractions showed enrichments of 370 ± 82.8 FC and 29 ± 12 FC, respectively, relative to unsorted controls.

To evaluate potential overlap with the glial lineage, we performed co-staining for Vimentin, a marker for Müller glia^12–14^(Fig. 6A). In the adult INL, we observed a complete absence of colocalization between Nectin-3 and Vimentin (Fig. 6A, upper right panel, insets). At P5, we observed a dynamic profile characterized by low or absent colocalization in the developing AC layer (Fig. 6A, middle right panel). In contrast, at E18, Nectin-3^+^ cells in the developing GCL showed strong colocalization with Vimentin, alongside distinct Vimentin-positive cells that remained negative for Nectin-3 (Fig. 6A, bottom right panel).

Transcriptional analysis of the Müller glia marker *Glul* (Fig. 6B) revealed no significant differences across most stages, with the exception of a significant downregulation in the adult triple-positive (0.13 ± 0.06 FC) and P5 single-positive (0.29 ± 0.09 FC) fractions. A significant increase in *Glul* expression was only observed in the E18 Nectin-3^+^/Sca-1^+^ subpopulation (2.08 ± 0.19 FC).

Finally, to complement the cross-sectional analysis, we evaluated retinal flat mounts for colocalization with the pan-neuronal marker βIII-Tubulin (Supplementary Figure S1). At E18, Nectin-3^+^ cells were relatively large, displaying a perinuclear immunoreactivity pattern that strongly overlapped with βIII-Tubulin. By P5, Nectin-3^+^ cells appeared smaller, with signal predominantly localized to the soma and a marked segregation from βIII-Tubulin. In the adult retina, Nectin-3^+^ some cells were significantly smaller than their embryonic counterparts but re-established strong colocalization with βIII-Tubulin within the GCL. Collectively, these results suggest that Nectin-3^+^ cells belong to a specific neuronal pool within the retina rather than the glial scaffold.

## Discussion

This study identifies Nectin-3 as a novel surface marker that characterizes a molecularly distinct cell population in the mouse retina across embryonic, postnatal, and adult stages. Our sorting strategy enabled the isolation of specific subpopulations based on the co-expression of Nectin-3, CD117, and Sca-1, providing a reliable foundation for gene expression analysis. While previous research has established Nectin-3 as a critical adhesive component in the ciliary body^8^ and its disruption has been linked to congenital cataracts and anterior retinal inversion^15^, its specific role as a marker for neuronal subpopulations within the neural retina has not been previously described. We demonstrate that these Nectin-3^+^ cells are transcriptionally enriched for markers typically associated with early retinal development, including *Nestin*,* Pax6*,* Sox2*,* Chx10*, and *Rax*. Notably, the persistence of a rare Nectin-3^+^ population in the adult retina suggests that these cells retain a molecular signature reminiscent of an undifferentiated state.

The transcriptional profile of Nectin-3^+^ cells shows notable parallels with the molecular signatures typically found in retinal progenitor cells (RPCs). Specifically, the enrichment of *Chx10* in adult triple-positive subsets is of particular interest, as this factor is an established regulator of neuroretinal identity and is characteristically expressed during early developmental stages^16–23^. To evaluate the functional state of Nectin-3^+^ cells, we assessed the expression of *Syp*, a key marker of presynaptic vesicles^24^. Across all developmental stages, Nectin-3^+^ subpopulations showed no significant differences in *Syp* expression relative to unsorted controls. Given that neuronal maturation is typically characterized by increased synaptic protein levels^25^ and specific axonal enrichment^26^, this lack of enrichment suggests these cells may remain in a developmentally “primed” state. While conclusions based on a single gene are inherently cautious, these results suggest that Nectin-3^+^ cells might possess a basal neuronal identity without the full transcriptional machinery required for functional integration into mature retinal circuits.

The spatiotemporal mapping and transcriptional profiling of Nectin-3^+^ cells provide compelling evidence that this population belongs to a specialized neuronal lineage, specifically associated with the GCL. Our immunohistochemical analysis across development showed a consistent localization of these cells within the forming and mature GCL, corroborated by the robust enrichment of the RGC marker *Rbpms*. This aligns with the established timeline of murine retinogenesis, where RGCs are the first neurons to be born (peaking between E11 and P0) and are already executing complex transcriptional programs for axon guidance by E15.5^27^.

A critical question arising from our findings was whether the identified Nectin-3^+^ population might represent a subset of Müller glia. Müller glia have been extensively characterized for their role in retinal homeostasis and their potential for neural regeneration^28–30^. These cells are typically identified by Vimentin expression and the localization of their somata within the inner nuclear layer (INL)^31^. Notably, Müller glia exhibit elevated expression of several transcription factors investigated in this study, including *Sox2*, *Chx10*, *Pax6*, and *Rax*^28,30,32,33^. Furthermore, CD117, which we utilized as a co-marker for subpopulation isolation, has previously been reported in Müller glia^34^. Our results definitively place Nectin-3^+^ cells within the neuronal lineage; while early embryonic Nectin-3^+^ cells (E18) showed some colocalization with Vimentin, this overlap was completely lost in the adult retina. Furthermore, the transcriptional levels of *Glul*—a definitive functional marker for Müller glia^35^—were either unchanged or significantly downregulated in adult Nectin-3^+^ fractions. This suggests that while these cells may share structural protein expressions with progenitors during the early waves of development, they ultimately diverge from the glial fate to establish a distinct neuronal identity. Perhaps the most striking finding is the decoupling of lineage commitment from functional maturation. While adult Nectin-3^+^ cells express *Rbpms* and βIII-Tubulin, they lack significant enrichment of the synaptic vesicle marker *Syp*, a “synaptic lag” that is particularly interesting given that inner retinal components often show a relative delay in achieving synaptic maturity compared to the outer retina^36^.

Our PCA data further underscores this, showing that adult triple-positive (Nectin-3^+^/CD117^+^/Sca-1^+^) cells remain transcriptomically “anchored” to an embryonic state, characterized by the persistent expression of *Nestin*, *Pax6*, *Rax*, and the neuroretinal regulator *Chx10*. Our analysis revealed a high degree of transcriptional similarity between the adult and embryonic (E18) populations, which clustered in close spatial proximity. In contrast, the postnatal (P5) Nectin-3^+^ subsets appeared as distinct outliers, likely reflecting a unique, transient transcriptional state during the peak of postnatal expansion. This biological shift is further evidenced by our morphological analysis; at P5, we observed only a few cells showing weak colocalization with RBPMS, while the majority did not colocalize. The transient loss of RBPMS colocalization at P5, coinciding with peak population expansion, suggests a developmentally regulated decoupling of lineage identity and maturation state, potentially reflecting a proliferative or transitional window. This reduction in neuronal marker expression at the peak of the population’s expansion is a classic hallmark of a transiently undifferentiated or proliferative state, suggesting that the P5 population represents a developmentally plastic window that warrants future investigation as a primary driver of retinal cell-fate dynamics. Rather than following the standard trajectory of RGC dendritic expansion and stratification that occurs between P1 and P13^37,38^, P5 Nectin-3^+^ cells maintain a high nuclear-to-cytoplasmic ratio. This unique combination of an RGC-like position, a “primed” transcriptional profile, and a lack of mature synaptic machinery suggests these cells may serve as a reservoir of early-born neurons that have bypassed terminal differentiation. While it remains to be determined if these cells represent a novel, undescribed retinal subpopulation or a distinct class of neurons, our findings align with recent evidence suggesting that certain adult RGC subtypes (e.g., C33/C40) retain transcriptional features of an embryonic cell state^39^. Together, these features indicate that Nectin-3^+^ cells represent a residual pool with molecular properties that warrant further investigation regarding their role in retinal plasticity.

Although rare, the persistence of Nectin-3^+^ cells in the adult retina is notable. The selective retention of an early-stage transcriptional program suggests that these cells preserve some of the core elements of a primitive molecular identity. The persistence of an embryonic-like transcriptional signature in adult neurons aligns with emerging concepts of ‘developmental memory’ and partial dedifferentiation states observed in other CNS compartments^40–43^, suggesting that such cells may represent a latent plasticity reservoir rather than terminally differentiated endpoints. This interpretation points toward a quiescent, immature state and underscores the necessity of investigating whether this population represents a functional reservoir of latent plasticity. Our findings provide a critical rationale for testing these theories, as the ability to prospectively isolate these cells now allows for a definitive assessment of their functional potential and their possible role in endogenous retinal repair.

Several markers have been proposed for neural or retinal progenitors, including CD133, CD117 (c-kit), Sca-1, and ABCG2 side-population assays. Each, however, presents distinct limitations: CD133 expression is highly heterogeneous across tissues; CD117 identifies multiple progenitor systems beyond the retina; and ABCG2-based dye efflux assays are technically demanding and often lack specificity. Furthermore, Sca-1 lacks a human ortholog. While this complicates translational comparisons, Sca-1 was utilized as an established model marker in this murine context, though Nectin-3 remains the central focus of this study. By contrast, Nectin-3 is an antibody-accessible cell surface protein with established roles in tissue organization. These features suggest that Nectin-3 provides a more specific and practical tool for the prospective isolation of cells with immature molecular profiles across all developmental stages. The ability to isolate viable Nectin-3^+^ populations via advanced sorting technologies offers a refined starting point for downstream functional studies, such as sorted-cell-derived organoids or neurosphere assays. Furthermore, the enrichment of *Pax6*, *Rax*, and *Chx10*—factors known to influence neuronal–glial fate^44–49^—provides a molecular rationale for exploring the intrinsic plasticity of these subsets in vitro. While long-term applications in cell-replacement strategies remain speculative, our results indicate that identifying and isolating these Nectin-3^+^ cells is a necessary step toward understanding retinal maintenance and the potential for endogenous repair.

Mechanistically, Nectin-3 operates as a Ca^2+^-independent adhesion molecule mediating homophilic and heterophilic interactions, including binding to CD155 and integration into integrin-rich domains^6,7^. By utilizing a polyclonal antibody targeting the C-terminal region (aa 431–549) exclusive to this isoform, we confirm that the observed signal represents the canonical alpha variant typically associated with afadin-binding and signaling pathways. This isoform likely facilitates niche interactions that support undifferentiated states by regulating cell polarity and environmental communication. Rather than a passive marker, Nectin-3 may coordinate signaling pathways like NOTCH, which regulates progenitor quiescence and fate decisions through force-dependent receptor-ligand interactions^50,51^. Since NOTCH signaling controls Nectin expression during ocular morphogenesis^52^, understanding how Nectin-3 interfaces with NOTCH, Hippo/YAP, and Wnt will be critical to explaining how immature molecular profiles are preserved in the adult retina^53–55^. These mechanisms suggest Nectin-3-mediated adhesion is a fundamental component of the niche supporting retinal cell plasticity.

Several limitations of this study should be considered. While transcriptomic enrichment characterizes the immature molecular profile of Nectin-3^+^ cells, the precise functional role of this population remains to be determined. Importantly, this study is designed as a prospective marker identification and characterization framework, rather than a functional lineage study. Establishing such markers is a prerequisite for downstream functional assays, which are currently not feasible without prior enrichment of the target population. While transcriptomic enrichment characterizes the immature molecular profile of Nectin-3^+^ cells, the precise functional role of this population remains to be determined. We acknowledge that the enrichment of markers like *Sox2* and *Nestin* can also be observed in mature or reactive glia^30,56^. Therefore, future studies utilizing BrdU/EdU proliferation assays or neurosphere assays to demonstrate self-renewal will be necessary to functionally confirm the progenitor-like nature of these cells. Future investigations utilizing lineage tracing, specialized in vitro assays, or single-cell RNA sequencing (scRNA-seq) will be essential to definitively reveal their identity and differentiate between a persistent progenitor state and a novel, quiescent neuronal subpopulation. While the colocalization with RBPMS and the significant enrichment of the *Rbpms* transcript, coupled with the lack of Vimentin staining and *Glul* expression, strongly suggest a commitment to a neuronal rather than a glial lineage, the functional significance of Nectin-3-mediated adhesion in the maintenance of these cells within the ganglion cell layer requires further mechanistic dissection. This study serves as a necessary characterization of a molecularly unique cell fraction, providing the groundwork for identifying its exact place within the established hierarchy of retinal cell classes.

In summary, this is the first study to identify Nectin-3 as a novel and practical surface marker for isolating specific retinal cell subpopulations across developmental stages and in adulthood. Our findings provide a strong molecular rationale for further investigation into these populations, as the ability to prospectively isolate Nectin-3^+^ cells now creates unique opportunities to dissect the programs governing retinal plasticity and latent regenerative potential. By establishing Nectin-3 as an accessible entry point for studying these rare cells, this work lays the foundation for future studies in disease modeling and the exploration of endogenous repair mechanisms in the mammalian retina.

## Methods

### Animals and ethical statement

Specific pathogen-free C57BL/6J wild-type mice were purchased from the Central Laboratory for Experimental Animals at the Medical University of Warsaw. Mice were housed in a dedicated facility on a 12-hour light/dark cycle with *ad libitum* access to standard chow and water. All animal procedures were performed in strict accordance with the European Act on the protection of animals used for scientific or educational purposes and were approved by the 2nd Local Ethics Committee for Animal Experimentation in Warsaw (Agreement No. WAW2/072/2024). All animal procedures and reporting complied with the ARRIVE guidelines.

### Retinal single-cell suspension

Retinas were isolated from C57BL/6J mice at three developmental stages: embryonic day 18 (E18), postnatal day 5 (P5), and adult (2–3 months old). Following euthanasia by CO₂ asphyxiation, eyes were immediately enucleated, and the retinas were dissected. The tissue was minced and transferred to a digestion buffer consisting of DMEM (Mediatech, Inc.), 336 U/ml collagenase IV (Worthington Biochemical), and 248 U/ml DNase I (Sigma-Aldrich). The tissue was incubated for 30 min at 37 °C in a humidified 5% CO₂ atmosphere and was gently triturated by pipetting every 10 min.

The enzymatic digestion was quenched by adding DMEM supplemented with 10% Fetal Bovine Serum (FBS). The cell suspension was then centrifuged at 344 x g for 10 min at room temperature. After discarding the supernatant, the cell pellet was washed once and finally resuspended in fresh DMEM for subsequent staining.

### Flow cytometry staining and sorting

The retinal single-cell suspension was stained for fluorescence-activated cell sorting (FACS). The cell pellet was resuspended in 50 µl of DMEM and incubated for 15 min at room temperature with a cocktail of the following primary antibodies: PE-conjugated anti-Ly-6 A/E (Sca-1) (BD Biosciences, Cat. No. 553108), APC-H7-conjugated anti-CD117 (c-kit) (BD Biosciences, Cat. No. 560185), Biotin-conjugated anti-Nectin3 (Antibodies online, Cat. No. ABIN715393).

Following primary antibody incubation, the cells were washed, and the pellet was resuspended in 50 µl of DMEM containing FITC-conjugated Streptavidin (BD Biosciences, Cat. No. 554060). This secondary incubation was performed for 15 min at room temperature, protected from light, to label the biotinylated Nectin3 antibody.

After a final wash, the cells were resuspended in a sorting buffer composed of Ca^2+^/Mg^2+^-free PBS with 0.05% BSA. To enable dead cell exclusion, the viability dye 7-Aminoactinomycin D (7-AAD) was added to the cell suspension immediately prior to analysis. Cell sorting was performed on a MoFlo Astrios EQ cell sorter (Beckman Coulter, Brea, CA, USA).

### Flow cytometry data analysis and statistics

After gating for single cells and excluding non-viable cells using 7-AAD, the size of each distinct cell population (e.g., Nectin-3^+^, Nectin-3^+^/CD117^+^) was calculated and expressed as a percentage of the total viable cell population.

All statistical analyses were performed in R (version 4.4.1). The percentages of the different cell populations were compared using a one-way analysis of variance (ANOVA), after confirming that the data met the assumptions of normality and homogeneity of variances. The cell population type served as the independent variable, and its percentage was the dependent variable. Following a significant ANOVA result (*p* < 0.05), a Tukey’s Honest Significant Difference (HSD) post-hoc test was conducted to identify specific pairwise differences between groups. A significance level of α = 0.05 was used for all statistical tests.

### RNA isolation and cDNA synthesis

Total RNA was extracted from sorted cell populations and age-matched unsorted control cells using the RNeasy Mini Kit (Qiagen, Cat. No. 74104) according to the manufacturer’s protocol. The concentration and purity of the isolated RNA were determined using spectrophotometry. Subsequently, total RNA was reverse transcribed into complementary DNA (cDNA) using the iScript™ cDNA Synthesis Kit (Bio-Rad, Cat. No. 170–8840).

### Quantitative PCR (qPCR)

Gene expression analysis was performed by quantitative PCR (qPCR) using the iTaq™ Universal SYBR^®^ Green Supermix (Bio-Rad, Cat. No. 1725124). All qPCR reactions were run in triplicate. The primers used for amplifying the target genes and the *GAPDH* housekeeping gene are listed in Table 1.

**Table 1:** Primer sequences used for qPCR analysis.

Gene	Forward Primer (5’ → 3’)	Reverse Primer (5’ → 3’)
*Nestin*	CCTCAACCCTCACCACTCTATTTT	GCTTTTTACTGTCCCCGAGTTCTC
*Chx10*	GCCCACCTTCTTGGAAGTGCT	TGTGTCGCCGCTTCTTACGC
*Pax6*	CACCACACCTGTCTCCTCCT	ATAACTCCGCCCATTCACTG
*Sox2*	TAGAGCTAGACTCCGGGCGATGA	TTGCCTTAAACAAGACCACGAAA
*Rax*	CCCTGAGGCTAAACTTGCAG	GTTCCCTTCTCCTCCTCCAC
*GAPDH*	ACTCCACTCACGGCAAATTC	TCTCCATGGTGGTGAAGACA
*Rbpms*	AACGCTTTGAACGGCATCC	GGGCTACTGGGGTAAAGTGC
*Glul*	GTTCCCACTTGAACAAAGGCA	ACCCAGATATACATGGCTTGGA
*Syp*	AGTACCCATTCAGGCTGCAC	CCGAGGAGGAGTAGTCACCA

Gene expression was quantified using the cycle threshold (Cq) values, and the relative expression of each target gene was calculated using the delta-delta Ct (ΔΔCt) method. First, the Cq value for each target gene was normalized to that of the *GAPDH* housekeeping gene to obtain a ΔCq value (ΔCq=Cq_target_−Cq_GAPDH_). The ΔCq of each experimental sample was then normalized to the ΔCq of a designated control group (calibrator) to yield the ΔΔCq value (ΔΔCq = ΔCq_sample_ − ΔCq_control_). To ensure normalization rigor across developmental stages, the control group for each age (E18, P5, and adult) consisted of a matching number of cells obtained from the corresponding unsorted retinal cell suspension. The fold change in gene expression, relative to the control group, was subsequently calculated using the formula 2^−ΔΔCq^. Results are presented as the mean fold change ± standard error of the mean (SEM) from at least three independent biological replicates.

Statistical analysis was performed using R software (version 4.4.1). A one-way ANOVA, followed by a HSD post-hoc test, was used to compare gene expression levels between cell populations. A p-value of less than 0.05 was considered statistically significant.

To explore the relationships among the various cell populations and time points, we conducted a principal component analysis (PCA) on the mean gene expression data. The analysis included all eight genes (*Nestin*,* Sox2*,* Pax6*,* Chx10*, *Rax*,* Syp*,* Rbpms* and *Glul*) and the Nectin-3^+^ cell populations, excluding the control conditions. Gene expression values, presented as relative expression change (fold change), were used as the input for the PCA. The resulting plot was used to visually identify clusters and trends among the samples, with the first three principal components (PC1, PC2, and PC3) used to visualize the primary axes of variation in three-dimensional space.

### Immunofluorescence and confocal imaging

Mice were euthanized by cervical dislocation, and then transcardially perfused with isotonic saline followed by 4% paraformaldehyde (PFA). Eyes were enucleated, post-fixed in 4% PFA for 2 h, and the retinas were carefully dissected. For cryosectioning, the eyes were cryoprotected in a graded sucrose series (10%, 20%, and 30% in PBS), embedded in Optimal Cutting Temperature (OCT) compound (Tissue-Tek), and frozen at − 80 °C. Radial retinal cross-Sect.  (14 μm) were obtained using a Leica cryostat (Leica Biosystems) and collected on Superfrost Plus slides. For immunostaining, sections were blocked and permeabilized for one hour at room temperature in a buffer containing 2.5% donkey serum and 0.5% Triton X-100 in PBS. Tissues were then incubated overnight at 4 °C with a cocktail of primary antibodies: rabbit anti-βIII-Tubulin (1:500; Abcam, ab18207) or rabbit anti-RBPMS (1:500; Abcam, ab152101) or mouse anti-Vimentin (1:500; Abcam, ab8978) and rat anti-Nectin-3 (1:300; Antibodies online, Cat. No. ABIN715393). After several washes in PBS, the retinas were incubated for two hours at room temperature, protected from light, with the corresponding secondary antibodies: Alexa Fluor 647-conjugated donkey anti-rabbit (1:1000; Thermo Fisher Scientific, A-31573) or Alexa Fluor 647-conjugated donkey anti-mouse (1:1000; Thermo Fisher Scientific, A-31571) and FITC-conjugated anti-rat (Antibodies online, Cat. No. ABIN715393). Following final washes, slides were mounted using an anti-fade mounting medium containing DAPI. Images were acquired using an LSM710 laser-scanning confocal microscope (Zeiss), and subsequent image processing was performed using ZEN blue software (Zeiss).

## Supplementary Information

Below is the link to the electronic supplementary material.

Supplementary Material 1

## Data Availability

The datasets generated and analyzed during the current study are available from the corresponding authors, Agnieszka Lukomska (agnieszka.lukomska@wum.edu.pl) or Magdalena Kucia (magdalena.kucia@wum.edu.pl), on reasonable request.

## References

[1] Ahmad, S. S., Blair, K. & Kanukollu, V. M. Optic Atrophy. in StatPearls (StatPearls Publishing, Treasure Island (FL), (2024). 32644556

[2] Allison, K., Patel, D. & Alabi, O. Epidemiology of Glaucoma: The Past, Present, and Predictions for the Future. *Cureus*10.7759/cureus.11686 (2020). doi:10.7759/cureus.11686. 33391921 10.7759/cureus.11686 PMC7769798

[3] Rikitake, Y., Mandai, K. & Takai, Y. The role of nectins in different types of cell–cell adhesion. *J. Cell. Sci.* **125**, 3713–3722 (2012). 23027581 10.1242/jcs.099572

[4] Sakisaka, T., Ikeda, W., Ogita, H., Fujita, N. & Takai, Y. The roles of nectins in cell adhesions: cooperation with other cell adhesion molecules and growth factor receptors. *Curr. Opin. Cell. Biol.* **19**, 593–602 (2007). 17942295 10.1016/j.ceb.2007.09.007

[5] Takai, Y., Irie, K., Shimizu, K., Sakisaka, T. & Ikeda, W. Nectins and nectin-like molecules: Roles in cell adhesion, migration, and polarization. *Cancer Sci.* **94**, 655–667 (2003). 12901789 10.1111/j.1349-7006.2003.tb01499.x PMC11160195

[6] Satoh-Horikawa, K. et al. Nectin-3, a New Member of Immunoglobulin-like Cell Adhesion Molecules That Shows Homophilic and Heterophilic Cell-Cell Adhesion Activities. *J. Biol. Chem.* **275**, 10291–10299 (2000). 10744716 10.1074/jbc.275.14.10291

[7] Mueller, S. & Wimmer, E. Recruitment of Nectin-3 to Cell-Cell Junctions through trans-Heterophilic Interaction with CD155, a Vitronectin and Poliovirus Receptor That Localizes to αvβ3 Integrin-containing Membrane Microdomains. *J. Biol. Chem.* **278**, 31251–31260 (2003). 12759359 10.1074/jbc.M304166200

[8] Inagaki, M. et al. Roles of cell-adhesion molecules nectin 1 and nectin 3 in ciliary body development. *Development* **132**, 1525–1537 (2005). 15728677 10.1242/dev.01697

[9] Umemoto, T. et al. Limbal Epithelial Side-Population Cells Have Stem Cell–Like Properties, Including Quiescent State. *Stem Cells*. **24**, 86–94 (2006). 16150918 10.1634/stemcells.2005-0064

[10] Kusanagi, R. et al. Nectin-3 expression is elevated in limbal epithelial side population cells with strongly expressed stem cell markers. *Biochem. Biophys. Res. Commun.* **389**, 274–278 (2009). 19716807 10.1016/j.bbrc.2009.08.130

[11] Dorgau, B. et al. Single-cell analyses reveal transient retinal progenitor cells in the ciliary margin of developing human retina. *Nat. Commun.* **15**, 3567 (2024). 38670973 10.1038/s41467-024-47933-x PMC11053058

[12] De Reis, M., Cabral-da-Silva, R. A., De Mello, M. E. C., Taylor, J. S. H. & F. G. & Müller glia factors induce survival and neuritogenesis of peripheral and central neurons. *Brain Res.* **1205**, 1–11 (2008). 18353289 10.1016/j.brainres.2008.02.035

[13] Okada, M., Matsumura, M., Ogino, N. & Honda, Y. Muller cells in detached human retina express glial fibrillary acidic protein and vimentin. *Graefes Arch. Clin. Exp. Ophthalmol.* **228**, 467–474 (1990). 2227494 10.1007/BF00927264

[14] Zayas-Santiago, A. et al. Unidirectional Photoreceptor-to-Müller Glia Coupling and Unique K+ Channel Expression in Caiman Retina. *PLoS ONE*. **9**, e97155 (2014). 24831221 10.1371/journal.pone.0097155 PMC4022631

[15] Lachke, S. A. et al. The cell adhesion gene PVRL3 is associated with congenital ocular defects. *Hum. Genet.* **131**, 235–250 (2012). 21769484 10.1007/s00439-011-1064-z PMC3279124

[16] Bachleda, A. R., Pevny, L. H. & Weiss, E. R. *Sox2* -Deficient Müller Glia Disrupt the Structural and Functional Maturation of the Mammalian Retina. *Investig Opthalmology Vis. Sci.* **57**, 1488 (2016). 10.1167/iovs.15-17994 PMC4819558 27031842

[17] Dhomen, N. S. et al. Absence of *Chx10* Causes Neural Progenitors to Persist in the Adult Retina. *Investig Opthalmology Vis. Sci.* **47**, 386 (2006). 10.1167/iovs.05-0428 PMC2423807 16384989

[18] Dorval, K. M., Bobechko, B. P., Ahmad, K. F. & Bremner, R. Transcriptional Activity of the Paired-like Homeodomain Proteins CHX10 and VSX1. *J. Biol. Chem.* **280**, 10100–10108 (2005). 15647262 10.1074/jbc.M412676200

[19] Horsford, D. J. et al. *Chx10* repression of *Mitf* is required for the maintenance of mammalian neuroretinal identity. *Development* **132**, 177–187 (2005). 15576400 10.1242/dev.01571

[20] Lin, Y., Ouchi, Y., Satoh, S. & Watanabe, S. Sox2 Plays a Role in the Induction of Amacrine and Müller Glial Cells in Mouse Retinal Progenitor Cells. *Investig Opthalmology Vis. Sci.* **50**, 68 (2009). 10.1167/iovs.07-1619 18719084

[21] Ma, W., Yan, R. T., Li, X. & Wang, S. Z. Reprogramming Retinal Pigment Epithelium to Differentiate Toward Retinal Neurons with *Sox2*. *Stem Cells*. **27**, 1376–1387 (2009). 19489100 10.1002/stem.48 PMC2746631

[22] Thiel, G. How Sox2 maintains neural stem cell identity. *Biochem. J.* **450**, e1–e2 (2013). 23445224 10.1042/BJ20130176

[23] Wang, Z., Yasugi, S. & Ishii, Y. Chx10 functions as a regulator of molecular pathways controlling the regional identity in the primordial retina. *Dev. Biol.* **413**, 104–111 (2016). 27001188 10.1016/j.ydbio.2016.03.023

[24] Wiedenmann, B. & Franke, W. W. Identification and localization of synaptophysin, an integral membrane glycoprotein of Mr 38,000 characteristic of presynaptic vesicles. *Cell* **41**, 1017–1028 (1985). 3924408 10.1016/s0092-8674(85)80082-9

[25] Agholme, L., Lindström, T., Kågedal, K., Marcusson, J. & Hallbeck, M. An In Vitro Model for Neuroscience: Differentiation of SH-SY5Y Cells into Cells with Morphological and Biochemical Characteristics of Mature Neurons. *J. Alzheimers Dis.* **20**, 1069–1082 (2010). 20413890 10.3233/JAD-2010-091363

[26] Li, L. et al. Visualizing the Distribution of Synapses from Individual Neurons in the Mouse Brain. *PLoS ONE*. **5**, e11503 (2010). 20634890 10.1371/journal.pone.0011503 PMC2901335

[27] Lo Giudice, Q., Leleu, M., La Manno, G. & Fabre, P. J. Single-cell transcriptional logic of cell-fate specification and axon guidance in early born retinal neurons. *Development dev.* **178103**10.1242/dev.178103 (2019). 10.1242/dev.178103 31399471

[28] Fischer, A. J. & Reh, T. A. Müller glia are a potential source of neural regeneration in the postnatal chicken retina. *Nat. Neurosci.* **4**, 247–252 (2001). 11224540 10.1038/85090

[29] Guimarães, R. P. D. M. et al. Evidence of Müller Glia Conversion Into Retina Ganglion Cells Using Neurogenin2. *Front. Cell. Neurosci.* **12**, 410 (2018). 30483060 10.3389/fncel.2018.00410 PMC6240664

[30] Surzenko, N., Crowl, T., Bachleda, A., Langer, L. & Pevny, L. SOX2 maintains the quiescent progenitor cell state of postnatal retinal Müller glia. *Development* **140**, 1445–1456 (2013). 23462474 10.1242/dev.071878 PMC3596988

[31] Goel, M. & Dhingra, N. K. bFGF and insulin lead to migration of Müller glia to photoreceptor layer in rd1 mouse retina. *Neurosci. Lett.* **755**, 135936 (2021). 33910061 10.1016/j.neulet.2021.135936

[32] Furukawa, T. et al. Hes1, and notch1 Promote the Formation of Müller Glia by Postnatal Retinal Progenitor Cells. *Neuron* **26**, 383–394 (2000). 10839357 10.1016/s0896-6273(00)81171-x

[33] Yoshimoto, T. et al. The Rax homeoprotein in Müller glial cells is required for homeostasis maintenance of the postnatal mouse retina. *J. Biol. Chem.* **299**, 105461 (2023). 37977220 10.1016/j.jbc.2023.105461 PMC10714373

[34] Too, L. K., Gracie, G., Hasic, E., Iwakura, J. H. & Cherepanoff, S. Adult human retinal Müller glia display distinct peripheral and macular expression of CD117 and CD44 stem cell-associated proteins. *Acta Histochem.* **119**, 142–149 (2017). 28110937 10.1016/j.acthis.2016.12.003

[35] Roesch, K. et al. The transcriptome of retinal Müller glial cells. *J. Comp. Neurol.* **509**, 225–238 (2008). 18465787 10.1002/cne.21730 PMC2665263

[36] Olney, J. W. An electron microscopic study of synapse formation, receptor outer segment development, and other aspects of developing mouse retina. *Invest. Ophthalmol.* **7**, 250–268 (1968). 5655873

[37] Coombs, J. L., Van Der List, D. & Chalupa, L. M. Morphological properties of mouse retinal ganglion cells during postnatal development. *J. Comp. Neurol.* **503**, 803–814 (2007). 17570502 10.1002/cne.21429

[38] Diao, L., Sun, W., Deng, Q. & He, S. Development of the mouse retina: Emerging morphological diversity of the ganglion cells. *J. Neurobiol.* **61**, 236–249 (2004). 15389605 10.1002/neu.20041

[39] Rheaume, B. A. et al. Pten inhibition dedifferentiates long-distance axon-regenerating intrinsically photosensitive retinal ganglion cells and upregulates mitochondria-associated Dynlt1a and Lars2. *Development* **150**, dev201644 (2023). 10.1242/dev.201644 PMC10163351 37039265

[40] Blasco-Chamarro, L. & Fariñas, I. Fine-tuned Rest: Unveiling the Regulatory Landscape of Adult Quiescent Neural Stem Cells. *Neuroscience* **525**, 26–37 (2023). 37437796 10.1016/j.neuroscience.2023.07.003

[41] Lugert, S. et al. Quiescent and Active Hippocampal Neural Stem Cells with Distinct Morphologies Respond Selectively to Physiological and Pathological Stimuli and Aging. *Cell. Stem Cell.* **6**, 445–456 (2010). 20452319 10.1016/j.stem.2010.03.017

[42] Meng, H. et al. Quiescent Adult Neural Stem Cells: Developmental Origin and Regulatory Mechanisms. *Neurosci. Bull.* **40**, 1353–1363 (2024). 38656419 10.1007/s12264-024-01206-1 PMC11365920

[43] Urbán, N., Blomfield, I. M. & Guillemot, F. Quiescence of Adult Mammalian Neural Stem Cells: A Highly Regulated Rest. *Neuron* **104**, 834–848 (2019). 31805262 10.1016/j.neuron.2019.09.026

[44] Canto-Soler, M. V., Huang, H., Romero, M. S. & Adler, R. Transcription factors CTCF and Pax6 are segregated to different cell types during retinal cell differentiation. *Dev. Dyn.* **237**, 758–767 (2008). 18224715 10.1002/dvdy.21420 PMC2443865

[45] Hsieh, Y. W. & Yang, X. J. Dynamic Pax6 expression during the neurogenic cell cycle influences proliferation and cell fate choices of retinal progenitors. *Neural Develop*. **4**, 32 (2009). 10.1186/1749-8104-4-32 PMC2741438 19686589

[46] Lu, F. et al. *Rax* Is a Selector Gene for Mediobasal Hypothalamic Cell Types. *J. Neurosci.* **33**, 259–272 (2013). 23283339 10.1523/JNEUROSCI.0913-12.2013 PMC3582370

[47] Muranishi, Y., Terada, K. & Furukawa, T. An essential role for *Rax* in retina and neuroendocrine system development. *Dev. Growth Differ.* **54**, 341–348 (2012). 22524605 10.1111/j.1440-169X.2012.01337.x

[48] Rezanejad, H. et al. In vitro differentiation of adipose-tissue-derived mesenchymal stem cells into neural retinal cells through expression of human PAX6 (5a) gene. *Cell. Tissue Res.* **356**, 65–75 (2014). 24562376 10.1007/s00441-014-1795-y

[49] Zabiegalov, O. et al. Generation of a Double Reporter mES Cell Line to Simultaneously Trace the Generation of Retinal Progenitors and Photoreceptors. *Cells* **14**, 252 (2025). 39996725 10.3390/cells14040252 PMC11854395

[50] Kopan, R. & Ilagan, M. X. G. The canonical Notch signaling pathway: unfolding the activation mechanism. *Cell* **137**, 216–233 (2009). 19379690 10.1016/j.cell.2009.03.045 PMC2827930

[51] Kovall, R. A., Gebelein, B., Sprinzak, D. & Kopan, R. The Canonical Notch Signaling Pathway: Structural and Biochemical Insights into Shape, Sugar, and Force. *Dev. Cell.* **41**, 228–241 (2017). 28486129 10.1016/j.devcel.2017.04.001 PMC5492985

[52] Pang, J. et al. NOTCH Signaling Controls Ciliary Body Morphogenesis and Secretion by Directly Regulating Nectin Protein Expression. *Cell. Rep.* **34**, 108603 (2021). 33440163 10.1016/j.celrep.2020.108603 PMC8422303

[53] Ding, R. & Berger, C. Hippo pathway regulates neural stem cell quiescence. *Cell. Cycle*. **15**, 1525–1526 (2016). 27058021 10.1080/15384101.2016.1171653 PMC4934065

[54] Duncan, A. W. et al. Integration of Notch and Wnt signaling in hematopoietic stem cell maintenance. *Nat. Immunol.* **6**, 314–322 (2005). 15665828 10.1038/ni1164

[55] Lampada, A. & Taylor, V. Notch signaling as a master regulator of adult neurogenesis. *Front. Neurosci.* **17**, 1179011 (2023). 37457009 10.3389/fnins.2023.1179011 PMC10339389

[56] Motoyoshi, A., Saitoh, F., Iida, T. & Fujieda, H. Nestin Regulates Müller Glia Proliferation After Retinal Injury. *Investig Opthalmology Vis. Sci.* **64**, 8 (2023). 10.1167/iovs.64.14.8 PMC10631512 37934159

