# Supplementary material for: Spatiotemporal characterization of a distinct nectin-3+ cell population in the developing and adult mouse retina
Source: Sci Rep. 2026 Jun 8;16:26088. doi: 10.1038/s41598-026-56219-9 (PMC13490412; doi:10.1038/s41598-026-56219-9)
Supplement: Supplementary file 1 [file 41598_2026_56219_MOESM1_ESM.pdf]

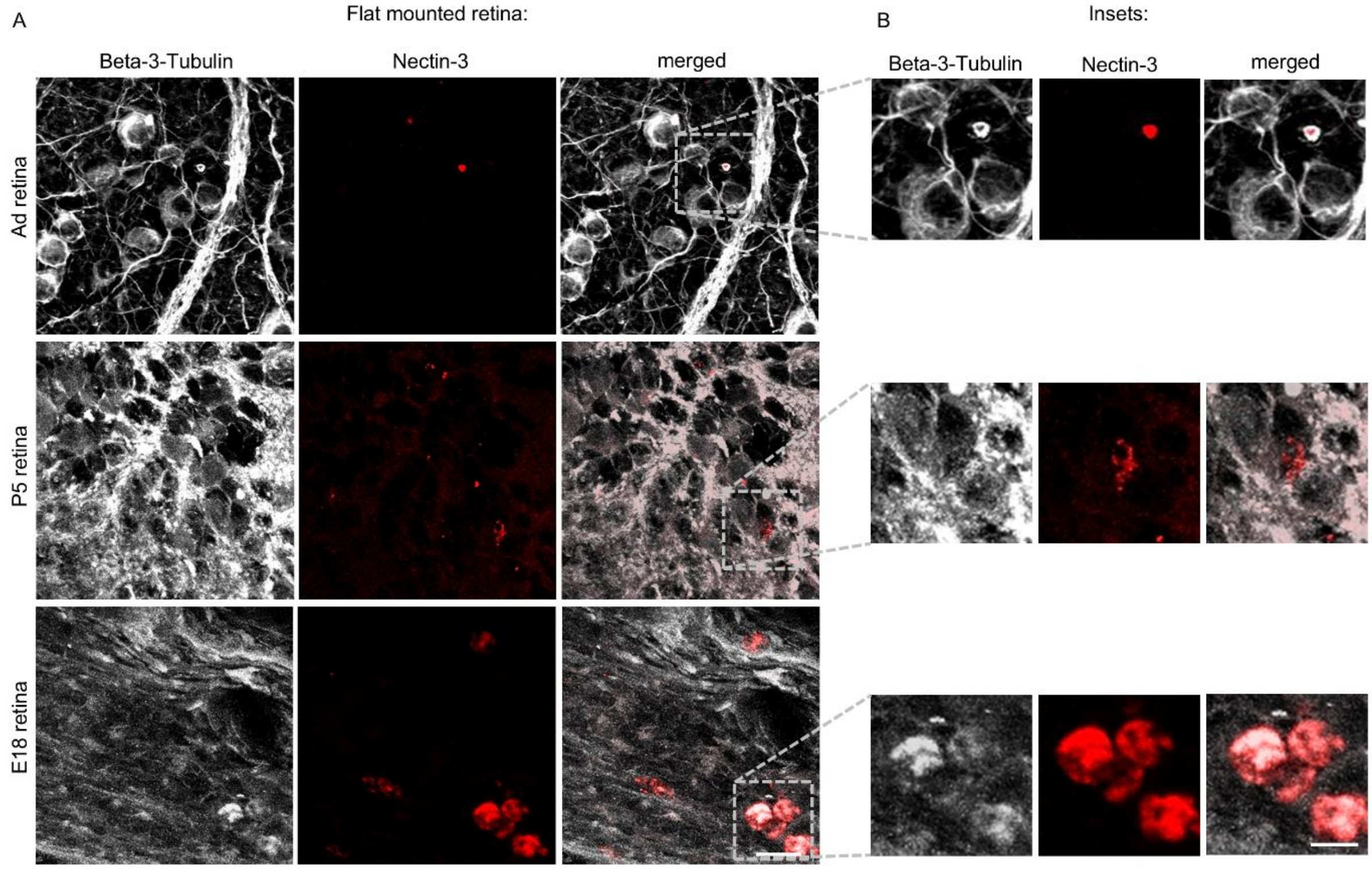

Supplementary Figure 1. Morphological and localization characteristics of Nectin-3⁺ cells in retinal flat mounts. Representative confocal microscopy images of retinal flat mounts from E18, P5, and adult mice. Tissues were immunostained for Nectin-3 (red) and the pan-neuronal marker βIII-Tubulin (grayscale). (A) Representative images show the general distribution and morphology of Nectin-3⁺ cells at each developmental stage. Scale bar: 20 µm. (B) Higher-magnification insets of the indicated regions highlight the colocalization of Nectin-3 and βIII-Tubulin at E18 and in adult retinal ganglion cells, which is absent at P5. Scale bar: 10 µm.
